# Clinical Application of Droplet Digital PCR for Hepatitis Delta Virus Quantification

**DOI:** 10.3390/biomedicines10040792

**Published:** 2022-03-29

**Authors:** Antonella Olivero, Chiara Rosso, Alessia Ciancio, Maria Lorena Abate, Aurora Nicolosi, Giulia Troshina, Angelo Armandi, Davide Giuseppe Ribaldone, Giorgio Maria Saracco, Elisabetta Bugianesi, Mario Rizzetto, Gian Paolo Caviglia

**Affiliations:** 1 Department of Medical Sciences, University of Turin, 10100 Turin, Italy; chiara.rosso@unito.it (C.R.); alessia.ciancio@unito.it (A.C.); marialorena.abate@unito.it (M.L.A.); aurora.nicolosi@unito.it (A.N.); ytroshina@cittadellasalute.to.it (G.T.); angelo.armandi@unito.it (A.A.); davidegiuseppe.ribaldone@unito.it (D.G.R.); giorgiomaria.saracco@unito.it (G.M.S.); elisabetta.bugianesi@unito.it (E.B.); mario.rizzetto@unito.it (M.R.); gianpaolo.caviglia@unito.it (G.P.C.); 2 Division of Gastroenterology, Città della Salute e della Scienza–Molinette Hospital, 10100 Turin, Italy

**Keywords:** chronic hepatitis D, ddPCR, HDV

## Abstract

Droplet digital PCR (ddPCR) is a novel developed PCR technology providing the absolute quantification of target nucleic acid molecules without the need for a standard curve and regardless PCR amplification efficiency. Our aim was to develop a ddPCR assay for Hepatitis Delta virus (HDV)-RNA viremia quantification and then evaluate its performance in relation to real-time PCR methods. Primers and probe were designed from conserved regions of HDV genome to detect all the 8 HDV genotypes; the World Health Organization (WHO)-HDV international standard was used to calculate the conversion factor transforming results from copies/mL to IU/mL. To evaluate the clinical performance of ddPCR assay, plasma specimens of HDV-infected patients were tested and results were compared with data obtained with two real-time quantitative PCR (RT-qPCR) assays (i.e., in-house assay and commercial RoboGene assay). Analyzing by linear regression a series of 10-fold dilutions of the WHO-HDV International Standard, ddPCR assay showed good linearity with a slope coefficient of 0.966 and R^2^ value of 0.980. The conversion factor from copies to international units was 0.97 and the quantitative linear dynamic range was from 10 to 1 × 10^6^ IU/mL. Probit analysis estimated at 95% an LOD of 9.2 IU/mL. Data from the evaluation of HDV-RNA in routine clinical specimen of HDV patients exhibited strong agreement with results obtained by RT-qPCR showing a concordance correlation coefficient of 0.95. Overall ddPCR and RT-qPCR showed highly comparable technical performance. Moreover, ddPCR providing an absolute quantification method may allow the standardization of HDV-RNA measurement thus improving the clinical and diagnostic management of delta hepatitis.

## 1. Introduction

Hepatitis delta virus (HDV) is a defective RNA virus that requires the helper function of hepatitis B virus (HBV) for virion assembly and propagation [1]. World-wide, more than 350 million people are chronically infected by HBV and about 20 million of those are estimated to be also infected with HDV [2]. In HBV/HDV co-infected patients, HDV causes a severe liver disease with an accelerating course of progression toward cirrhosis and an increased risk of hepatocellular carcinoma [3].

The first step in the diagnosis of HDV infection is the detection of total antibodies against the hepatitis delta antigen (anti-HD). To assess whether the presence of anti-HD reflects an ongoing active HDV infection or represents a serologic marker of a past HDV infection, testing for HDV-RNA in serum is mandatory. HDV-RNA viral load quantification is a direct gauge of viral replication, essential to diagnose an active HDV infection and to monitor treatment response in patients undergoing antiviral therapy [4]. 

At present, real time quantitative PCR (RT-qPCR) is the method commonly used to evaluate HDV-RNA viral load and until now several in-house and commercial assays have been developed. Unfortunately, as shown by Le Gal et al. in the First International External Quality Assessment for HDV-RNA quantification [5], results from different laboratories showed a high variability due to the heterogeneity of technical tools as extraction methods, RT-qPCR devices, internal control, and quantification standard employed. Discrepant performances were more evident in the quantification of samples other than from genotype 1, probably because of the genetic heterogeneity of HDV genotypes and of the presence of mismatches to the viral genomic target present in primers and probe sequences [6]. Indeed, Stelzl et al. highlighted the importance of introducing a correction factor, based on the World Health Organization (WHO) international standard for HDV-RNA, to improve the comparability of quantitative results obtained in different laboratories, using the same commercial assay but employing different nucleic acid extraction protocols and amplification/detection devices [7].

Droplet digital PCR (ddPCR) is a recently developed third generation PCR technology that enables the absolute quantification of target nucleic acid molecules. Coupling microfluidic technology and emulsion chemistry, samples are partitioned into up to 20,000 nanoliter-sized droplets, with each supporting single end-point PCR amplification. Poisson algorithms, from the ratio of positive to total partitions, are used to calculate the absolute number of target nucleic acid in the original sample [8]. This emerging technology exhibited significant improvements compared to RT-qPCR such as: absolute quantification without the need for a standard curve and regardless PCR amplification efficiency, low susceptibility to PCR inhibitors, and high sensibility and reproducibility [9]. 

Starting from first digital PCR applications [10,11,12], several different ddPCR commercial platforms have been developed based on different technical strategies to achieve sample dispersion and signal counting [13,14,15]. Currently, research and clinical ddPCR usages range from copy number variation detection [16] to gene expression analysis [17], rare cancer targets detection [18], and quantitation of pathogens [19,20].

In this study, we developed a new assay for HDV-RNA viremia quantification employing ddPCR technology and we evaluated its clinical diagnostic utility by comparing its performance to an in-house RT-qPCR assay routinely used in our laboratory and the commercial assay RoboGene HDV RNA quantitation 2.0 (Analytik Jena, Jena, Germany).

## 2. Materials and Methods

### 2.1. Patient Specimens and Viral RNA Extraction

Plasma specimens of HDV-infected patients, obtained from residual material originally collected for clinical testing, were used in this study. All samples derived from a cohort of patients with chronic Delta hepatitis followed at the division of Gastroenterology, A.O.U. Città della Salute e della Scienza–Molinette Hospital of Turin. To conduct the longitudinal study, sequential plasma samples of HDV patients who underwent antiviral therapies were evaluated.

Viral RNA was extracted from 400 µL of plasma using the EZ1 DSP virus kit on the EZ1 Advanced XL instrument (QIAGEN GmbH, Hilden, Germany) following manufacturer’s recommendations. An RNA internal control (RQC-RNA Quality Control Kit–BIOCLARMA, Turin, Italy) was added to each sample to monitor the extraction procedure, pointing out the presence of inhibitory effects and reducing the risk of false-negative results. Extracted RNA was eluted in 60 µL of elution buffer and stored at −20 °C until analysis. 

### 2.2. Primers and Probe Design

Primers and probe were designed using Primer3Plus software (https://primer3plus.com, accessed on 14 November 2016) from conserved regions of aligned sequences of 8 different HDV genotypes, relying on HDV complete genome, NCBI Reference Sequence: NC_001653, retrieved from GenBank. The same set of primers and probe was used both for RT-qPCR and for ddPCR application. Sequence and position of primers and probe were: primer Forward HDVA1 5′ TCTCCCTTAGCCATCCGAGT 3′ (818–837), primer Reverse HDVA2 5′ CGTCCTTCTTTCCTCTTCG 3′ (902–920), and Probe 5′ 6FAM ATGGCATCTCCACCTCCTC Iowa Black FQ 3′ (876–894).

### 2.3. WHO-HDV International Standard

The first WHO-HDV international standard (PEI code 7657/12, Paul-Ehrlich Institute, Langen, Germany), recently developed through an international collaborative study coordinated by Paul–Erlich Institute [21] was used to calibrate both methods and to calculate the conversion factor transforming results from copies/mL to IU/mL. The standard has been prepared using a genotype 1 strand of HDV derived from HDV-positive human plasma, further diluted in human negative plasma, and finally supplied as lyophilized material. A series of 10-fold dilutions of the WHO-HDV International Standard was used to calculate the percentage of recovery.

### 2.4. RT-qPCR

One-step RT-qPCR was performed in a final volume of 20 µL. For each amplification reaction 5 µL of purified RNA was added to a reaction mixture containing 10 µL of iTaq Universal Probes One-Step reaction mix (BIO-RAD Laboratories, Hercules, CA, USA), 0.5 µL of iScript Reverse Transcriptase, 1 µL of HDV primers/FAM labeled probe (900 nm/250 nm) mix 20×, 1 µL of RQC primers/VIC labeled probe mix 20× (RQC-RNA Quality Control Kit—BIOCLARMA, Turin, Italy), and PCR-grade H_2_O up to the final volume. The amplification process was performed using the CFX96 Real-Time System BIO-RAD (BIO-RAD Laboratories, Hercules, CA, USA) according to the following thermal cycling conditions: hold 10 min at 50 °C for reverse transcription reaction, 3 min at 95 °C for polymerase activation and DNA denaturation, 45 cycles of denaturation at 95 °C for 15 s and annealing /extension + plate reading at 62 °C for 30 s. The assay was a multiplex RT-qPCR using two different reporter fluorophores: FAM for HDV-RNA target and VIC for RNA internal control. The standard curve was generated by plotting the mean cycle threshold (Ct) values against a 10-fold serial dilution of a cDNA standard quantified in international units (IU) against the WHO-HDV international standard. 

### 2.5. Droplet Digital PCR

Droplet digital PCR was performed on the Bio-Rad’s QX200 Droplet Digital PCR system (BIO-RAD Laboratories, Hercules, CA, USA), using the One-Step RT-ddPCR Advanced Kit for Probes (BIO-RAD Laboratories, Hercules, CA, USA), according to manufacturer’s instructions. The reaction mixture consisted of 5.5 µL of One-Step RT-ddPCR Supermix, 2.2 µL Reverse Transcriptase, 1.1 µL DTT (100 mM), 1.1 µL of HDV primers/FAM labeled probe (900 nm/250 nm) mix 20×, 0.55 µL of RQC primers/VIC labeled probe mix 20× (RQC-RNA Quality Control Kit –BIOCLARMA, Torino, Italy), 0.55 µL of PCR-grade H_2_O, and 11 µL of extracted RNA in a final volume of 22 µL. A total of 20 µL of each reaction mix were loaded into a sample well of a DG8 cartridge and after the addition of 70 µL droplet generation oil, droplets were generated by a QX200 droplet generator (BIO-RAD Laboratories, Hercules, CA, USA). The DG8 cartridge is composed of three rows consisting of eight wells each. The bottom wells are filled with the oil, the middle wells are loaded with the sample reaction mix and in the top wells are collected the droplets after generation. Droplets were subsequently transferred to a single 96-well plate (Eppendorf, Hamburg, Germany) and PCR amplification was performed on a T100 Thermal Cycler (BIO-RAD Laboratories, Hercules, CA, USA). To aspirate droplets, it is fundamental to hold the pipet tip at a 15 °C angle against the bottom edge of the sample well and to pipet slowly to avoid creating air bubbles. Moreover, during droplets transfer it is recommended to cover the 96-well plate with 8-cap strips to prevent evaporation and contamination with particulates and between wells. The PCR thermal profile was set up as follows: hold 60 min at 50 °C for reverse transcription reaction, 10 min at 95 °C for enzyme activation, 40 cycles of denaturation at 95 °C for 30 s and annealing/extension at 61 °C for 1 min (ramp rate 2–3 °C/s), hold 10 min at 98 °C for enzyme deactivation and final hold at 4 °C. After PCR amplification, droplet fluorescence was analyzed by QX200 Droplet Reader (BIO-RAD Laboratories, Hercules, CA, USA), a two-channel detection system. Data were analyzed by QuantaSoft Software (BIO-RAD Laboratories, Hercules, CA, USA) and results were expressed as copies/µL of PCR reaction.

### 2.6. Optimization of Annealing Temperature of ddPCR Assay

To assess the optimal annealing temperature, an annealing thermal gradient was performed. The plate was run in a T100 Thermal Cycler according to the standard ddPCR protocol replacing the annealing temperature step with an annealing thermal gradient between 64 and 58 °C.

### 2.7. Statistical Analysis

Statistical analysis was performed using MedCalc software, version 18.0. (MedCalc, Ostend, Belgium). The coefficient of determination (R^2^), calculated by linear regression analysis, was used to characterized both methods; analytical sensitivity was assessed carrying out a Probit regression analysis for the estimation of the limit of detection (LOD). The two assays were compared by Deming regression analysis and by non-parametric Passing and Bablock regression [22]. The Bland-Altman method was used to test assays agreement [23]. For all the analysis, a *p* value < 0.05 was considered statistically significant. 

## 3. Results

### 3.1. Assessment of Optimal Annealing Temperature

**Figure 1:**
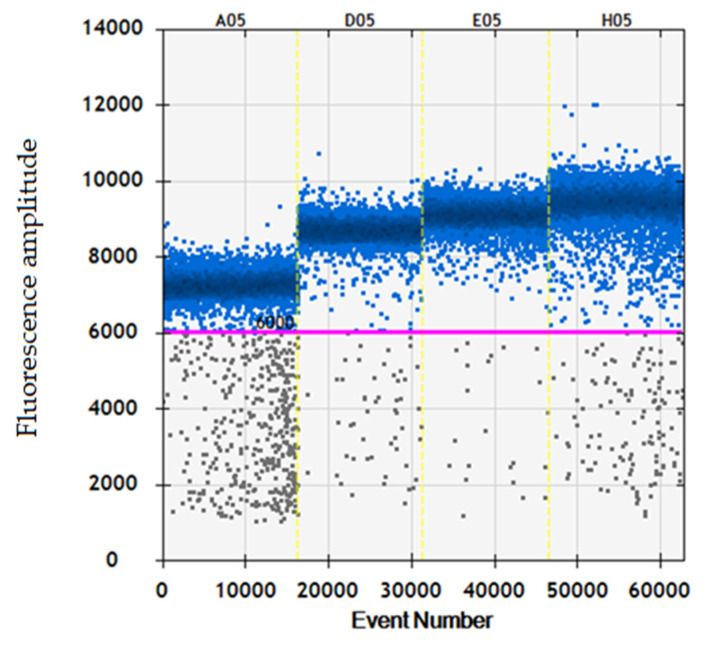
Fluorescence intensity plotted against annealing temperature gradient from 64 to 58 °C of a ddPCR assay for the quantitative determination of HDV-RNA in plasma samples. Annealing temperature were 64 °C in well A05, 61.7 °C in well D05, 60.3 °C in well E05, and 58 °C in well H05. The horizontal red line represents the threshold which establish a cut-off in the fluorescence intensity signal to differentiate between positive and negative droplets in a ddPCR assay. Each column depicts the negative (black) and positive (blue) events for single ddPCR well. Abbreviations: channel (Ch).

Performing the annealing thermal gradient, we identified the temperature showing the higher partition in fluorescence intensity between positive and negative droplets. This temperature value was also associated with the lower amount of rain, assumed as the presence of droplets with a fluorescence intensity falling between the positive and negative droplet populations. An annealing temperature of 61 °C was selected for ddPCR assay (Figure 1).

### 3.2. ddPCR Threshold Setting and Internal Control

To differentiate between positive and negative droplets in a ddPCR assay, it is essential to establish a cut-off in the fluorescence intensity signal. The QuantaSoft software, supplied by the Bio-Rad ddPCR system, defines for each sample an individual fluorescence threshold above which all droplets with greater fluorescence intensity are considered as positive. By using this method, the threshold is generally set very close to the negative droplets’ population with the consequence that a great number of rain droplets are classified as positive.

**Figure 2:**
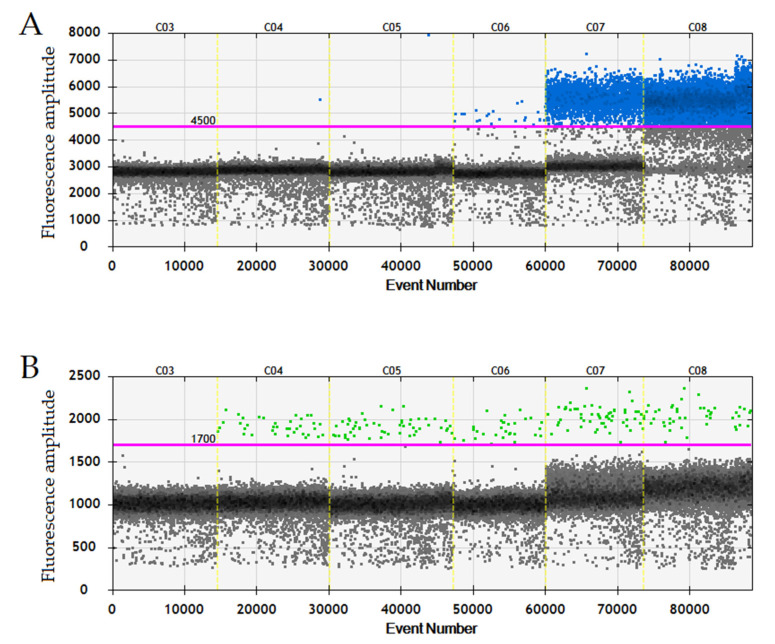
ddPCR experiment showing the quantitative determination of HDV-RNA in channel 1 (FAM) (**A**) and the RNA Internal Control in channel 2 (VIC) (**B**). Well C03 is the no template control.

With the purpose to set a global threshold to avoid bias between samples and to exclude false-positive results represented by droplets with an intermediate fluorescence signal too close to the threshold line, we used the manual threshold global (MTg) approach [24]. This method is based on the fluorescence amplitude observed in repeated no-template controls and negative samples and the same threshold, applied to all samples, is calculated as the average fluorescence signal of negative samples plus 6-fold the standard deviation. To monitor the whole procedure and to exclude false negative results, an RNA internal control was included starting from the RNA extraction step (Figure 2).

### 3.3. Conversion Factor Copies/mL to IU/mL, Linearity and Dynamic Range

The conversion factor from copies/mL to international units (IU/mL) and the linearity of RT-qPCR and ddPCR assays were determined by linear regression, analyzing a series of 10-fold dilutions of the WHO-HDV International Standard, starting from a concentration of 5.76 Log_10_ IU/mL. The estimated conversion factor from copies/mL to IU/mL was 1.01 for RT-qPCR and 0.97 for ddPCR (Table 1). 

**Figure 3:**
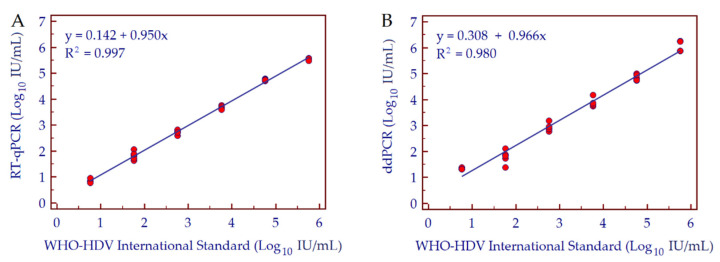
Scatter plot with regression lines between nominal and measured concentration of a 10-fold dilution series of WHO-HDV international standard using real time qPCR (**A**) and ddPCR (**B**). Three replicates were performed per dilution. Abbreviations: hepatitis D virus (HDV); World Health Organization (WHO).

Both methods showed good linearity with an estimated slope coefficient of 0.95 and R^2^ of 0.997 for RT-qPCR, and a slope coefficient of 0.966 and R^2^ value of 0.980 for ddPCR (Figure 3). 

**Figure 4:**
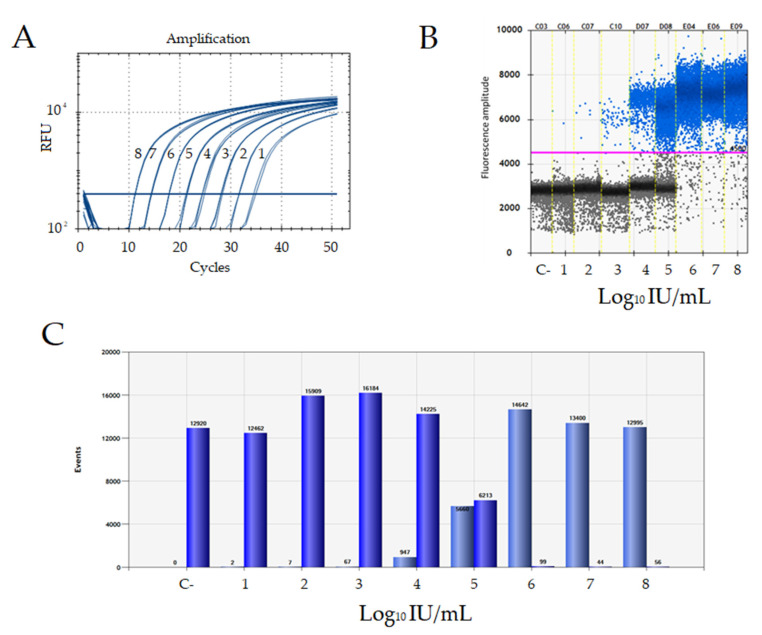
Determination of the assays linear range. (**A**) Amplification plot by RT-qPCR assay of a 10-fold dilution series (1 × 10^−1^ to 1 × 10^−8^) of a cDNA standard quantified in international units (IU) against the WHO-HDV international standard. Each dilution was tested in triplicate. Data are reported in Log_10_ scale. (**B**) One-dimensional plot of a ddPCR assay for the detection of a 10-fold dilution series (1 × 10^−1^ to 1 × 10^−8^) of a cDNA standard quantified in international units (IU) against the WHO-HDV international standard. Data are reported in Log_10_ scale. Individual droplets represent amplification of single or low copy number HDV-RNA template; blue droplets above the red threshold line are scored as positive, all black droplets below the red threshold line are scored as negative. Each column depicts the negative (black) and positive (blue) events for single ddPCR well. (**C**) ddPCR graphical representation of the droplet/events acquired by the droplet reader scored between positive droplet/events (light blue bars) and negative droplet/events (dark blue bars).

The linear range of both assays was evaluated by analyzing a 10-fold serial dilution, ranging from 1 × 10^−1^ to 1 × 10^−8^, of a cDNA standard calibrated against the WHO-HDV international standard. The linear range for RT-qPCR was from 1 × 10 to 1 × 10^8^ IU/mL (Figure 4A). ddPCR showed a quantitative linear dynamic range between 1 × 10 and 1 × 10^6^ IU/mL (Figure 4B); in fact, at the higher concentrations tested (i.e., 1 × 10^7^ and 1 × 10^8^ IU/mL) the reactions were saturated by an excess of target molecules and the low number of negative events detected didn’t enable the application of Poisson’s algorithms to calculate the number of copies in the starting sample (Figure 4C). Reported in Table 2 is the accuracy of recovery of a series of 10-fold dilutions of the WHO-HDV international standard tested with ddPCR. The mean recovery percentage was 101 ± 5%, suggesting a good recovery of HDV-RNA with minimal losses of template.

### 3.4. Analytical Sensitivity

**Figure 5:**
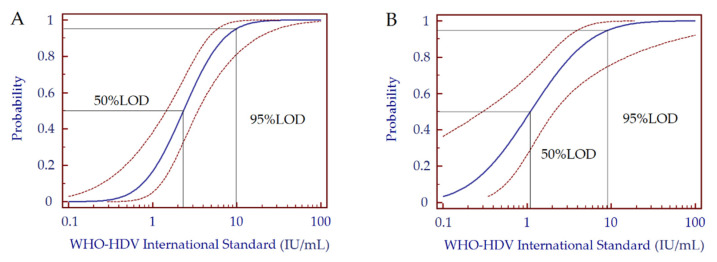
Probit analysis of HDV-RNA amplification data obtained by RT-qPCR assay (**A**) and ddPCR assay (**B**) for the estimation of the 95% and 50% limit of detection (LOD). Probit curves are depicted in blue; the two additional red dashed curves represent the 95% confidence interval for each probit curve. Abbreviations: hepatitis D virus (HDV); World Health Organization (WHO).

The analytical sensitivity of both RT-qPCR and ddPCR was determined by analyzing a 5-fold dilution series of the WHO-HDV international standard starting from 57.5 IU/mL (10^−4^ dilution) to 0.6 IU/mL (10^−6^ dilution) (Table 3). Probit analysis of HDV-RNA amplification data estimated for RT-qPCR indicated an LOD 95% of 9.7 (95%CI 5.8–29.7) IU/mL and an LOD 50% of 2.3 (1.4–3.4) IU/mL (Figure 5A); with the same analysis, ddPCR showed an LOD 95% of 9.2 (4.0–224.6) IU/mL and an LOD 50% of 1.1 (0.3–2.1) IU/mL (Figure 5B).

### 3.5. Precision and Specificity

To evaluate and compare the precision of RT-qPCR and ddPCR assays, 10-fold serial dilution ranging from 1 × 10 to 1 × 10^5^ of the WHO-HDV international standard was tested in various experiments and the coefficient of variation (CV) for each sample was calculated. The repeatability or intra-assay variability was evaluated by testing in triplicate each sample in the same experiment; to estimate the reproducibility or inter-assay variability, experiments were carried out on three different days (Table 4).

To determine the diagnostic specificity 10 HDV-negative specimens were tested and both assays showed no false positive results. To check for a possible cross-reactivity in samples from patients with viral coinfections, we analyzed 10 HBV and 10 HCV positive specimens with different viral loads and results obtained from both RT-qPCR and ddPCR assays were always negative (data not shown). In Table 4 principal technical features of RT-qPCR and ddPCR assays are summarized and compared.

### 3.6. Clinical Evaluation

**Figure 6:**
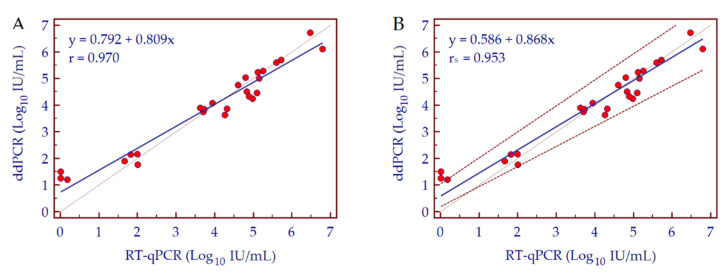
Regression analysis of HDV-RNA concentration values of clinical specimens obtained from HDV-infected patients, quantified by both RT-qPCR and ddPCR. (**A**) Deming regression analysis and (**B**) Passing and Bablok regression analysis.

**Figure 7:**
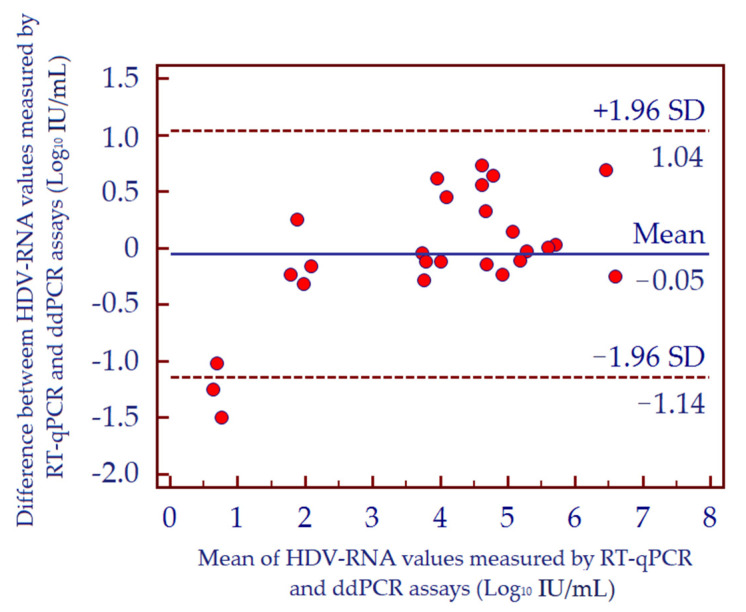
Bland-Altman plot comparing HDV-RNA values quantified by RT-qPCR and ddPCR assays. In the scatter diagram, the means of any two measurements shown in the *x* axis are plotted against the difference between those two measurements reported in the y axis. The blue horizontal line shows the mean of the differences and red horizontal dashed lines represent the 95% limits of agreement defined as the mean difference +1.96 SD of differences (1.04 and −1.14). Abbreviations: hepatitis D virus (HDV); standard deviation (SD).

To evaluate the diagnostic performance of HDV-RNA ddPCR assay, we analyzed 26 HDV-RNA positive patient samples with different viral load; data were compared with results obtained by RT-qPCR. At first, we calculated the concordance correlation coefficient that evaluates the degree of pairs of observations considering a measurement both of precision and accuracy of the analyzed data. The value of the concordance correlation coefficient was 0.95, suggesting a substantial strength of agreement between results obtained by the two assays. The concordance between the two methods was then assessed by Deming regression analysis in order to evaluate the variability of individual data measurements. Assays exhibited a high degree of correlation with a Pearson correlation coefficient (r) value of 0.970 (Figure 6A). The correlation of quantitative results from both tests was then assessed by Passing and Bablok regression analysis, showing a high concordance (Spearman rank correlation coefficient, r*_s_* = 0.953; *p* < 0.001). Moreover, the Cusum test for linearity, which determines if the residuals from the two sets of data are randomly distributed around the fitted line, showed no significant deviation from linearity (*p* = 0.530) (Figure 6B). Bland-Altman analysis also confirmed good agreement between the two assays; no significant bias was detected (−0.05 or −19.43%) (Figure 7).

**Figure 8:**
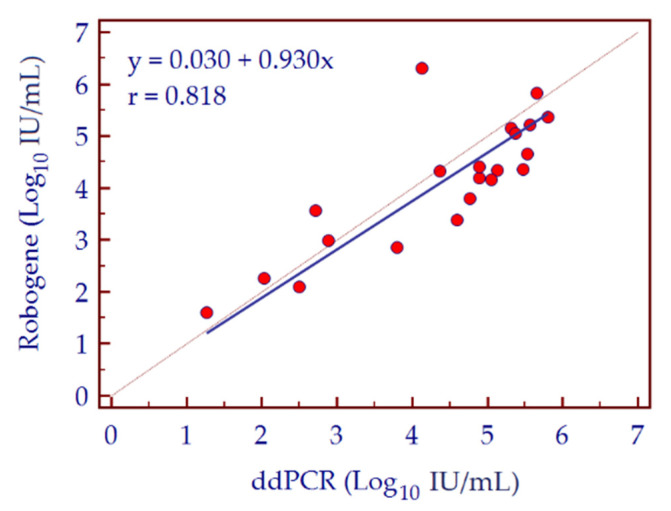
Deming regression analysis of HDV-RNA viral loads of 20 clinical specimens determined by ddPCR and RoboGene HDV RNA Quantification Kit 2.0.

**Figure 9:**
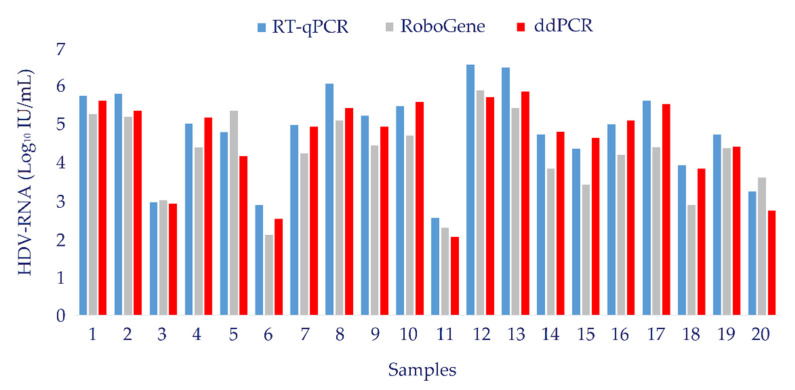
Histogram showing the comparison of HDV-RNA values obtained by RT-qPCR, ddPCR and RoboGene HDV RNA Quantification Kit 2.0. Blue bars represent values obtained by RT-qPCR. Data obtained by ddPCR and RoboGene HDV RNA assay are represented by red and gray bars, respectively.

Furthermore, HDV viral load values assessed by ddPCR in 20 specimens from a cohort of 20 HDV-infected patients were compared with data obtained from a commercial assay (RoboGene HDV RNA Quantification Kit 2.0, Analytik Jena, Jena, Germany). Results obtained by the two assays were comparable, as shown by Deming regression analysis resulting in a Pearson correlation coefficient value of 0.818 (Figure 8). Histograms in Figure 9 compared HDV-RNA viral loads obtained in the 20 specimens by RT-qPCR, ddPCR, and RoboGene HDV RNA assays.

**Figure 10:**
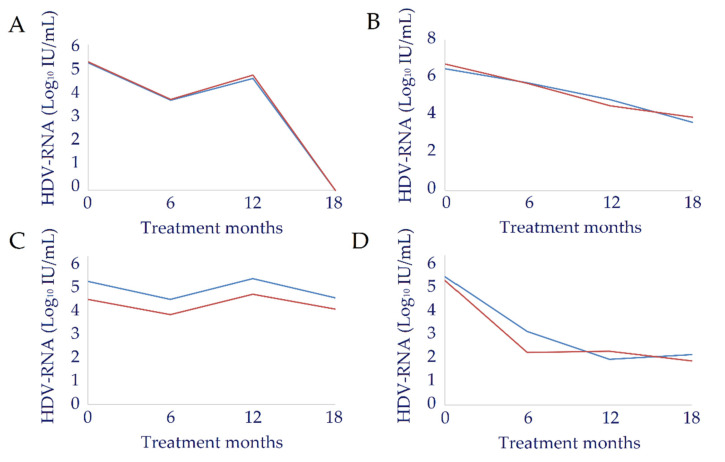
Comparison between kinetic patterns of HDV-RNA viral load measured by RT-qPCR (blue line) and ddPCR (red line) in longitudinal plasma samples from four patients (from **A**–**D**) treated with PEG-IFNα. HDV-RNA was evaluated at baseline and at months 6, 12 and 18 during treatment.

Finally, we conducted a longitudinal study evaluating sequential specimens of four patients that underwent antiviral therapy. Patients were treated with Pegylated Interferon alpha (PEG-IFNα) for 18 months and HDV-RNA was quantified at baseline and at months 6, 12, and 18 during treatment. Only one patient cleared HDV-RNA at the end of therapy; the other three patients were non-responders. As shown in Figure 10 data from RT-qPCR and ddPCR correlated well in all patients and at every time-point measurement resulting in very similar kinetic patterns of estimated HDV viral load.

## 4. Discussion

Hepatitis delta epidemiology, due to the implementation of vaccination programs against HBV, has significantly changed in the last decade showing a reduction of the prevalence of the infection in the global population. Nevertheless, hepatitis delta still remains a consistent medical burden both for its peculiar fast progression to liver cirrhosis and hepatocellular carcinoma and for the unsatisfactory therapeutic options currently available [25]. The quantification of HDV viral load is pivotal for monitoring treatment effectiveness and for more accurate clinical staging of chronic HDV infection. HDV viremia has in fact a significant impact on the disease progression, showing an association with a worse clinical outcome and being linked to an increased long–term risk for liver related events such as hepatic decompensation, hepatocellular carcinoma, or liver-related death/transplantation [26].

In this study, we have applied for the first time a new PCR technology, ddPCR, for the development and optimization of an assay for the quantitative determination of HDV-RNA as an alternative to standard RT-qPCR. 

The two assays shared a similar methodological approach using a one-step and a duplex reaction protocol with the addition of a synthetic RNA IC from the extraction step, which allows for monitoring the entire procedure, pointing out the presence of inhibitory effects and reducing the risk of false-negative results. Moreover, the addition of the reverse transcriptase enzyme to the master mix and the consequent possibility to perform a one-step protocol represents an advantage with respect to a two-step procedure because it allows the elimination of a separate step, thus reducing both the test execution time and the risk of sample contamination.

Another technical feature common to both our assays was the use of the same set of primers and probe, designed from conserved regions of aligned sequences of eight different HDV genotypes, originating from the spread of the delta virus genus. Different HDV genotype strains, most likely due to the selection of host immunological pressure and editing events, are distributed worldwide in specific geographical regions and are characterized by a high genetic variability which can reach up to 40% over the full genome sequence [6]. Targeting primers and probe of both assays on a specific HDV genome sequence with a high degree of conservation, such as the ribozyme region, increases the ability of the methods to detect and properly quantify samples from all different genotypes, avoiding the risk of underestimation or false negative results [5]. This is particularly significant in Italy and in other European countries, characterized by a concurrent decline of domestic infections and an increase of immigrants from countries in which hepatitis delta is still endemic [27].

Moreover, both assays were calibrated against the WHO-HDV international standard reference material, enabling the conversion of the results from copies/mL to IU/mL and allowing data standardization, and the possibility to compare results from different laboratories by employing different assays [21].

As expected, using the same set of primers and probes, the two assays showed highly comparable technical performances, exhibiting clearly similar LOD values. 

Comparing the reproducibility of the two methods, ddPCR showed superior repeatability and reproducibility, especially considering intra-assay measurements. The better performance exhibited by ddPCR is mainly due to the innovative features of this method. First, ddPCR, by providing an absolute quantification of target viral load, abrogates the need for a standard curve, conversely required in RT-qPCR assays, highly improving the homogeneity of results obtained from different laboratories. Indeed, calibrators used in different assays to generate a standard curve are very heterogeneous and can include different materials such as: synthetic HDV-RNA, HDV-cDNA plasmid, serum from HDV-infected patients or armored RNA. Moreover, RT-qPCR results may be influenced by the technique of quantification, batch, storage, and handling conditions of the calibrator used [4].

Furthermore, ddPCR data acquisition is based on end-point measurements. This feature makes ddPCR less dependent on the reaction efficiency and more tolerant to PCR inhibitors than RT-qPCR, where the fluorescence signal increases at each amplification cycle, proportionally to the amount of replicated DNA. The reduced sensitivity of ddPCR to PCR inhibitors makes this technique suitable also for rapid and low-cost quantifications of pathogens directly in the native specimen (i.e., blood, tissues, and stool) without the need of a previous step to perform nucleic acid extraction [28].

Owing to these peculiarities, the use of ddPCR in the management of viral infections has been growing steadily, showing to be a powerful diagnostic method. Until now, in the field of hepatitis virus, ddPCR assays have been developed for the detection of HBV [29,30,31], HEV [32] and HCV [33].

However, despite the listed advantages, ddPCR also presents some limits and open challenges. The relatively narrow dynamic range, 10 to 1 × 10^6^ IU/mL, if compared to RT-qPCR, 10 to 1 × 10^8^ IU/mL, is closely associated with the technical characteristics of the digital partition. A high target concentration leads to a saturation of positive droplets, making the Poisson statistic invalid and requiring, for samples with elevated viral load, the addition of an initial dilution step. Indeed, the BioRad ddPCR platform’s manufacturer recommends 1 µg of DNA per reaction as the sample input upper limit, while other platforms, such as the Rain-Dance ddPCR system, are able to analyze a larger amount of input DNA per reaction [34]. However, we estimated that the accuracy of recovery was 101%, thus suggesting an acceptable analytical error, and moreover negligible from a clinical point of view.

Another open issue in ddPCR assays is the threshold setting. ddPCR generates droplets that are classified as positive or negative referring to their position relative to a threshold settled to a certain fluorescence level; the determination of a correct threshold line is pivotal for reliable target quantification. However, setting the threshold is often complicated by the presence of droplets with an intermediate fluorescence, referred as “rain”, graphically positioned in an indeterminate region between the two distinct clusters of positive and negative droplets. It is still unclear if this intermediate fluorescence signal may be due to background fluorescence or to the presence of damaged positive droplets; nevertheless, a correct classification of rain droplets is critical, especially in samples with low target concentration. The QuantaSoft software supplied by BioRad offers an undisclosed method for an automated threshold assignment, which is not always accurate, but provides the possibility to choose alternatively a user-defined threshold setting. In this concern, several alternative methods have been developed by end-users in an attempt to standardize threshold determination. 

In the clustering method developed by Strain et al. [35], two thresholds are identified to delineate the two populations of positive and negative droplets, while rain droplets are discarded and excluded from the calculus of concentration. However, this clustering algorithm may generate an underestimation of the real concentration because a percentage of rain droplets results from suboptimal PCR reactions due to the presence of primers or probe mismatches that can occur when the target shows sequence variations. A similar approach was proposed by Jones et al. [36], called “definetherain”, and based on an open-access, freely available web-based JavaScript program. 

In our work, we adopted the manual threshold global (MTg) analysis proposed by Dreo et al. [24], which identifies the threshold as the averaged fluorescence signal in the no template controls (NTCs) and in negative samples plus 6-fold the standard deviation. With this approach the threshold set reflects the behavior of the individual assay and all droplets with fluorescence above the fixed threshold are considered positive. This analysis is expected to be more sensitive because it takes into account some droplets with intermediate fluorescence due to suboptimal PCR reactions, which are most likely present in our assay, considering the sequence variations that characterize the eight HDV genotypes.

Finally, ddPCR is currently more time-consuming, involving more samples transfer steps and more expensive than RT-qPCR but certainly hereafter, automation will help to make ddPCR competitive regarding these characteristics as well.

In our study, to prove the performance of ddPCR in clinical diagnostics, we tested clinical routine-derived specimens from a cohort of patients with chronic delta hepatitis, characterized by different viral loads. The statistical analysis showed a strong correlation with data obtained by RT-qPCR, also confirmed in a longitudinal study carried out by examining serial samples of HDV patients treated with PEG-IFNα for an 18-month period. In a small number of specimens, we observed greater variability, but always less than 1 Log_10_ IU/mL, most likely due to the presence in serum samples of inhibitors or interfering substances that can affect, mostly in RT, the PCR process. Moreover, we observed strong agreement between results achieved by the ddPCR assay and data obtained with a commercial kit for HDV-RNA quantification, RoboGene HDV RNA Quantification Kit 2.0, based on the RT-qPCR technique. 

## 5. Conclusions

In conclusion, ddPCR technology is a highly valuable and powerful method feasible for accurate quantification of HDV-RNA. In the management of patients with chronic HDV infection, the evaluation of HDV viremia using a reliable and sensitive assay is pivotal not only to predict the long-term clinical outcome of the disease, but also to monitor therapy effectiveness, and to predict post-treatment virological relapse [37,38]. Given the elevated specificity and sensitivity, and the possibility to directly quantify HDV-RNA without the need for a standard curve, our ddPCR assay may allow the standardization of HDV-RNA measurement and consequently improve the clinical and diagnostic management of delta hepatitis.

## Figures and Tables

**Table 1:** Determination of the conversion factor from copies/mL to IU/mL for RT and ddPCR assays.

HDV WHO (5.76 Log_10_ IU/mL)	RT-qPCR HDV-RNA (Log_10_ Copies/mL)	ddPCR HDV-RNA (Log_10_ Copies/mL)
10^−1^	5.71	5.86
10^−2^	5.66	5.96
10^−3^	5.73	6.00
Mean	5.70	5.94
Conversion factor (copies/mL to IU/mL)	1.01	0.97

Numbers in columns 2 and 3 represent the amount of HDV-RNA in Log_10_ copies/mL after correction for the dilution shown in column 1, while “Mean” is the mean of the three values. Abbreviations: hepatitis D virus (HDV); World Health Organization (WHO).

**Table 2:** ddPCR accuracy of recovery.

HDV WHO (5.76 Log_10_ IU/mL) Dilutions	Expected HDV-RNA Concentration (Log_10_ IU/mL)	Measured HDV-RNA Concentration (Log_10_ IU/mL)	% Recovery
10^−1^	4.76	4.67	98.1
10^−1^	4.76	4.59	96.4
10^−1^	4.76	4.83	101.5
10^−2^	3.76	3.69	98.1
10^−2^	3.76	3.69	98.1
10^−2^	3.76	4.05	107.7
10^−3^	2.76	2.74	99.3
10^−3^	2.76	2.81	101.8
10^−3^	2.76	3.10	112.3

The measured HDV-RNA concentration is the amount of HDV-RNA in Log_10_ IU/mL after correction for dilution. Abbreviations: hepatitis D virus (HDV).

**Table 3:** Probit regression data.

Dilution Factor	HDV-RNA (IU/mL)	RT-qPCR Replicates (*n*)	RT-qPCR Positive (*n*)	ddPCR Replicates (*n*)	ddPCR Positive (*n*)
10^−4^	57.5	9	9	7	7
10^−4.5^	18.2	9	9	8	8
10^−5^	5.8	20	16	8	7
10^−5.5^	1.8	13	7	11	7
10^−6^	0.6	12	0	9	3

Abbreviations: hepatitis D virus (HDV).

**Table 4:** Comparison of technical features between RT-qPCR and ddPCR.

Technical Features	RT-qPCR	ddPCR
Conversion Factor: Copies/mL to IU/mL	1.01	0.97
Quantification range (IU/mL)	1 × 10^−1^ × 10^8^	1 × 10^−1^ × 10^6^
Sensitivity: 95% Limit of Detection (IU/mL)	9.7	9.2
Sensitivity: 50% Limit of Detection (IU/mL)	2.3	1.1
Repeatability: intra-assay (% CV)	1.42	0.80
Reproducibility: inter-assay (% CV)	1.78	1.51

Abbreviations: coefficient of variation (CV); hepatitis D virus (HDV); World Health Organization (WHO).

## Data Availability

The data presented in this study are available upon request from the corresponding author.
